# Special Issue “Current Research on the Role of the Gut Microbiota in Human Diseases and Health”

**DOI:** 10.3390/ijms27093804

**Published:** 2026-04-24

**Authors:** Sanda Maria Cretoiu

**Affiliations:** Department of Morphological Sciences, Cell and Molecular Biology and Histology Discipline, Carol Davila University of Medicine and Pharmacy, 050474 Bucharest, Romania; sanda.cretoiu@umfcd.ro

The gut microbiota is increasingly understood as not merely a collection of commensal microorganisms, but as a dynamic ecological network embedded within host physiology [1,2]. Beyond compositional diversity, emerging research highlights the importance of functional integration, adaptive buffering capacity, and network stability in maintaining systemic homeostasis [2,3]. In this evolving landscape, the focus of microbiome science is shifting from the descriptive cataloging of taxa toward understanding how microbial ecosystems respond to environmental perturbations, sustain resilience, or transition toward destabilization [4].

Rather than viewing dysbiosis as a static imbalance, current evidence suggests that microbial disruption may represent a threshold-dependent ecological shift characterized by loss of functional redundancy, impaired host–microbe feedback loops, and amplified inflammatory signaling [5]. Such a perspective necessitates integrative frameworks capable of linking molecular mechanisms, systemic regulatory networks, and clinical phenotypes within a unified systems biology model [1,6]. The field has moved beyond cataloging microbial taxa toward understanding dynamic ecosystem behavior under conditions of stress, adaptation, and collapse [7,8], supported by longitudinal stability analyses and emerging evidence for alternative states and sharp transitions in gut microbial communities [9].

The Special Issue was structured to integrate conceptual reviews with original research contributions and, as such, brings together four state-of-the-art reviews and three original research articles, intentionally balancing conceptual synthesis with empirical validation. The reviews provide integrative analyses across molecular reprogramming, redox signaling, neurobiological regulation, and oncologic therapy modulation, thereby establishing the mechanistic scaffolding of host–microbe interactions. Complementing these perspectives, the original research contributions extend the discussion into developmental and translational contexts, examining ecosystem disruption, probiotic restoration, and vaccine-oriented strategies in pediatric populations. The thematic structure reflects the multidimensional scope of current microbiome research—bridging theory and experiment, mechanism and phenotype, perturbation and resilience. In this sense, the thematic organization of the Special Issue parallels the conceptual progression from molecular regulation to ecosystem dynamics and ultimately to clinical expression—a trajectory that aligns with the resilience-based framework discussed in this Editorial, and therefore, the individual contributions within this collection collectively illustrate this conceptual trajectory.

## 1. Microbiota-Driven Molecular Reprogramming and Redox Signaling

The first conceptual cluster focuses on microbiota-mediated molecular regulation.

Rubas et al. (contribution 1) provide a comprehensive synthesis of how microbial metabolites influence DNA and RNA methylation, histone modification, and chromatin accessibility. The review integrates one-carbon metabolism, short-chain fatty acids, and epigenetic enzymatic pathways, positioning the gut microbiota as the driver of epigenomic plasticity. Importantly, this work also expands the microbiome field toward system-level gene regulatory mechanisms, emphasizing the microbiota-dependent phenotypic modulation.

**Figure 1:**
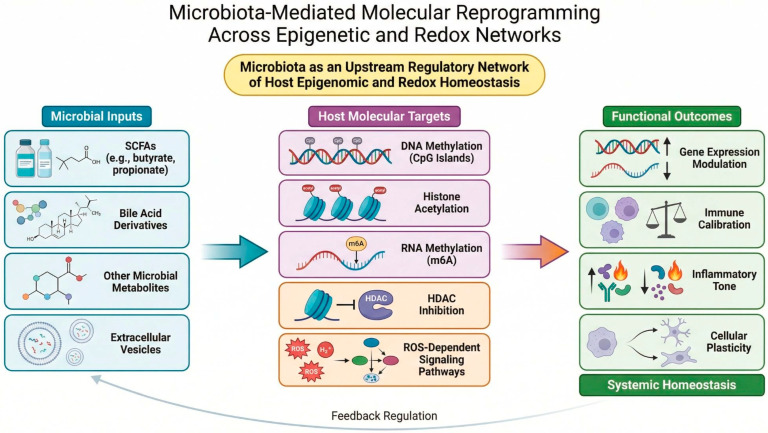
Microbiota-driven molecular regulatory interactions. The schematic illustrates how microbial metabolites and extracellular vesicles influence host epigenetic regulation and redox balance. Short-chain fatty acids, bile acid derivatives, and microbial signaling molecules interact with DNA methylation pathways, histone modification systems, and ROS-dependent signaling cascades, thereby modulating transcriptional programs and immune calibration at the molecular level.

Complementing this perspective, Ma et al. (contribution 2) explore the interplay between dysbiosis and oxidative imbalance, with their review highlighting extracellular vesicles as intercellular mediators linking microbial perturbations to inflammatory cascades and chronic disease pathways. By integrating redox biology with vesicle-mediated signaling, the authors frame oxidative stress as a microbiota-modulated systemic phenomenon rather than a localized biochemical event (Figure 1).

## 2. Gut–Brain Axis and Neurobiological Therapeutics

The neurobiological dimension of host–microbe interaction is addressed by Li et al. (contribution 3). This review synthesizes the mechanistic links among microbial composition, neurotransmitter regulation, circadian rhythm stability, and neuroinflammation, reflecting bidirectional communication between the gut microbiota and the central nervous system through neural, immune, endocrine, and barrier-mediated mechanisms. Microbial metabolites—including short-chain fatty acids and tryptophan derivatives, as well as cytokines and immune mediators—influence vagal signaling, systemic peptide hormones, and blood–brain barrier integrity. These pathways modulate neurotransmitter balance, circadian rhythm stability, and neuroinflammatory responses, thereby affecting sleep regulation.

By discussing probiotic and microbiota-targeted interventions, the authors shift the field toward therapeutic translation within the gut–brain axis framework, reinforcing the concept that sleep disturbances and neurobehavioral alterations may be modulated through microbial ecosystem interventions.

The model highlights the gut–brain axis as an integrated regulatory network in which microbial ecosystem dynamics interact with neurophysiological processes, contributing to both adaptive homeostasis and pathological dysregulation.

## 3. Microbiota and Oncologic Therapy Modulation

The systemic impact of microbiota perturbation during cancer therapy is analyzed by Guevara-Ramírez et al. (contribution 4). This review examines how chemotherapy, antibiotics, and immunotherapeutic regimens alter the microbial equilibrium and influence immune competence, treatment toxicity, and therapeutic responsiveness. The authors provide molecular insight into microbiota-mediated modulation of treatment efficacy, highlighting the microbiome as both a vulnerability factor and a potential supportive therapeutic target in hematologic malignancies.

These findings illustrate how therapeutic perturbations may act as ecological stress tests, revealing latent vulnerabilities in microbial network stability.

Cancer therapies significantly influence the gut microbial ecosystem, as chemotherapy, antibiotics, and immunotherapy induce microbial ecosystem disruption characterized by dysbiosis and reduced diversity. These alterations contribute to immune dysregulation, epithelial barrier impairment, and inflammatory amplification, which in turn influence treatment toxicity and therapeutic efficacy.

The model emphasizes the microbiome as a key determinant of therapeutic responsiveness and highlights feedback mechanisms through which microbial composition may modulate clinical outcomes. Within a systems framework, cancer therapy is both a driver and a consequence of microbial ecosystem instability, underscoring the importance of microbiota-informed strategies in oncologic management.

## 4. Pediatric Microbiota Development, Infection, and Therapeutic Intervention

A distinct cluster of original research contributions focuses on pediatric microbiota dynamics and translational strategies.

Murphy et al. investigate microbiota development and inflammatory markers in children following pull-through surgery (contribution 5), providing clinical and molecular evidence that persistent dysbiosis may contribute to ongoing gastrointestinal symptoms. By integrating microbiota profiling with inflammatory parameters, this work underscores the importance of ecological restoration in post-surgical pediatric populations.

Richmond et al. evaluated the probiotic intervention in infants, demonstrating effects on gastrointestinal and respiratory health (contribution 6). The study moves beyond associative microbiome analysis toward controlled therapeutic assessment, illustrating how specific strains may modulate immune responses and infection susceptibility during early life.

Salvador-Erro et al. contribute to vaccine development research through the use of outer membrane vesicles (OMVs) (contribution 7). While primarily centered on pathogen immunology, this study intersects microbiota research by addressing microbial–host immune interactions and mucosal immunity, highlighting the manner in which engineered bacterial systems may serve as vaccine platforms in the context of enteric infections and antibiotic resistance challenges.

These findings underscore the dynamic plasticity of the early-life intestinal microbial ecosystem and its role in immune education and barrier maturation. Following birth, microbial colonization shapes immune development and epithelial integrity during a critical window of immune programming. Post-surgical disruption may induce dysbiosis, persistent inflammatory activation, cytokine production, and reactive oxygen species (ROS) generation, contributing to increased infection susceptibility.

Probiotic intervention with *Bifidobacterium longum* subsp. *infantis* promotes the restoration of microbial complexity, regulatory T cell (Treg) expansion, and immune modulation, reducing infection risk (contribution 6). In parallel, outer membrane vesicle (OMV)-based strategies represent a platform for targeted mucosal immunity through activation of memory T and B cell responses (contribution 7).

The model emphasizes early-life microbial ecosystem plasticity as both a vulnerability and an opportunity for resilience-based therapeutic intervention.

## 5. Integrative Perspective and Conceptual Outlook

Across all contributions, a unifying principle emerges: the gut microbiota operates as a regulatory interface influencing epigenetic programming, redox balance, neurobiological function, immune competence, and therapeutic responsiveness. The diversity of topics represented in this Special Issue—from molecular epigenomics and oxidative signaling to pediatric dysbiosis, oncologic therapy modulation, and microbiota-targeted interventions—reflects the progressive maturation of the field.

Collectively, these works reinforce a systems biology perspective in which the gut microbiota should be understood as a dynamic ecological network embedded within host physiology [1,5]. Disease states increasingly appear not as isolated organ failures, but as manifestations of disrupted host–microbe network stability, and within this framework, dysbiosis represents a loss of ecosystem resilience—an impaired capacity to maintain functional equilibrium in the face of environmental, inflammatory, or therapeutic stressors [2].

Future progress in microbiome science will depend on integrating multi-omics technologies, longitudinal designs, and mechanistic validation models capable of distinguishing causality from correlation. Precision interventions targeting microbial composition, metabolite production, and immune calibration must be grounded in an ecological understanding of microbial adaptability and host feedback mechanisms.

Ultimately, the gut microbiota should be regarded as an integral component of systemic regulatory networks rather than merely a secondary disease modifier.

Here, one introduces the “Gut Microbiota Ecosystem Resilience Threshold Model,” a system-level framework that conceptualizes the gut microbiota as a dynamic ecological network operating at the interface of microbial functional redundancy and host regulatory integration. This model defines ecosystem stability not by taxonomic diversity alone, but by the capacity of interconnected microbial–host signaling networks to maintain adaptive buffering under sustained perturbation. Unlike traditional dysbiosis-centered frameworks that emphasize compositional imbalance, and beyond general ecological shift or tipping-point theories [10], the Gut Microbiota Ecosystem Resilience Threshold Model explicitly integrates (i) microbial functional redundancy, (ii) host–microbe feedback regulation, and (iii) molecular downstream reprogramming within a unified systems architecture. This resilience-oriented perspective is conceptually consonant with our recent Three-Layer Ecosystem Disruption Model (TLED) framework, which addressed how additive-rich ultra-processed dietary environments may erode gut ecosystem stability across structural, metabolic, and immune dimensions over the life course [11].

## 6. Structural Architecture of the Resilience Threshold Model

To enhance conceptual clarity, the Gut Microbiota Ecosystem Resilience Threshold Model can be operationally structured into four interconnected regulatory layers:

Layer I—Environmental Perturbation Inputs.

This layer comprises exogenous drivers capable of challenging ecosystem stability, including dietary patterns, infections, antibiotic exposure, oncologic therapies, environmental toxicants, and psychosocial stressors. These perturbations act as dynamic stress inputs whose magnitude, duration, and frequency determine system load.

Layer II—Microbial Functional Network.

At the core of the model lies the microbial ecosystem defined not solely by taxonomic composition, but by functional redundancy, metabolic flux capacity, and interspecies network connectivity. Key parameters include pathway-level redundancy (metagenomic functional overlap), metabolite production stability (e.g., short-chain fatty acids, bile acid derivatives), and resilience of microbial interaction networks under perturbation. These parameters may be operationalized through metagenomic pathway richness, metabolomic flux variance analysis, and ecological network stability metrics derived from longitudinal profiling.

Layer III—Host Regulatory Integration Nodes.

This layer represents host domains that are directly coupled to microbial signaling, including:

▪ Epithelial barrier integrity;
▪ Immune calibration and inflammatory tone;
▪ Redox homeostasis;
▪ Epigenetic regulatory plasticity;
▪ Neuro-immune communication pathways.

These domains function as coupled regulatory nodes whose destabilization can propagate non-linearly across adjacent systems.

Layer IV—Clinical Phenotypic Output.

The final layer reflects emergent systemic outcomes, ranging from maintained homeostasis to chronic inflammatory, metabolic, neurobehavioral, or oncologic phenotypes. Importantly, phenotypic manifestation is conceptualized as a late-stage output of cross-layer destabilization rather than a direct consequence of isolated microbial shifts.

## 7. Definition of the Threshold

Within this layered architecture, the resilience threshold is defined as a non-linear transition point at which:

1. Microbial functional redundancy falls below adaptive buffering capacity;
2. Host regulatory nodes exhibit synchronized destabilization across multiple domains.

Thus, the threshold does not represent a purely compositional tipping point, but a cross-domain failure of coupled microbial–host regulatory networks. The threshold is reached when reductions in microbial functional redundancy and destabilization of host regulatory nodes become mutually reinforcing.

While classical ecological resilience theory conceptualizes disturbance tolerance within self-contained ecosystems [12], the present framework introduces a dual-network instability model. Microbial destabilization is defined not solely by failure of compositional recovery, but by simultaneous dysregulation across host regulatory domains—including immune calibration, redox balance, epithelial integrity, and epigenetic modulation.

In contrast to descriptive microbiome transition models, which often focus on compositional state changes, this framework conceptualizes disease emergence as a non-linear failure of multi-level adaptive buffering across interconnected biological domains. Thus, the model bridges classical ecological resilience principles with molecular systems biology and clinical phenotype integration.

Within this framework, environmental drivers—including diet, infection, therapeutics, and environmental stressors—act as ecological perturbations that challenge microbial ecosystem stability. When buffering mechanisms and functional redundancy are preserved, the system maintains resilience, supports host regulatory integration, and sustains physiological homeostasis. However, once a critical tipping point is exceeded, ecosystem destabilization may occur, resulting in amplified inflammatory signaling, impaired host–microbe feedback loops, and maladaptive downstream effects across epigenomic, immune, neuro-immune, and redox networks. Importantly, exceeding this threshold should not be interpreted as necessarily implying absolute irreversibility; rather, it may indicate a state of heightened instability in which partial restoration of ecosystem functionality remains possible through timely and targeted interventions, including microbiota-directed, nutritional, or anti-inflammatory strategies.

## 8. Testable Implications of the Resilience Threshold Framework

The model generates experimentally testable implications. First, longitudinal multi-omics integration should reveal non-linear shifts in functional metabolic outputs (e.g., SCFA profiles, bile acid derivatives) preceding overt taxonomic collapse. Second, reduction in microbial functional redundancy is predicted to correlate more strongly with systemic inflammatory amplification than alpha-diversity indices alone. Third, combined indices integrating barrier integrity markers (e.g., tight junction proteins), circulating cytokine profiles, and microbial metabolite flux may serve as early indicators of proximity to ecological tipping points.

This threshold-based perspective integrates molecular mechanisms with systems biology principles, emphasizing that disease emergence may reflect a failure of ecosystem resilience rather than merely the presence of specific microbial taxa. By distinguishing adaptive perturbation from destabilizing transition, the model provides a conceptual bridge between microbial ecology, host regulatory networks, and clinical phenotypes.

To advance empirical validation, the proposed framework requires operationalization through quantifiable composite indices. Ecosystem resilience may be approximated by integrating (i) microbial functional redundancy metrics derived from metagenomic pathway analysis; (ii) quantitative metabolite flux profiling, including short-chain fatty acids and bile acid derivatives; (iii) epithelial barrier integrity markers such as tight junction protein expression; and (iv) systemic inflammatory tone assessed through circulating cytokine profiles.

**Figure 2:**
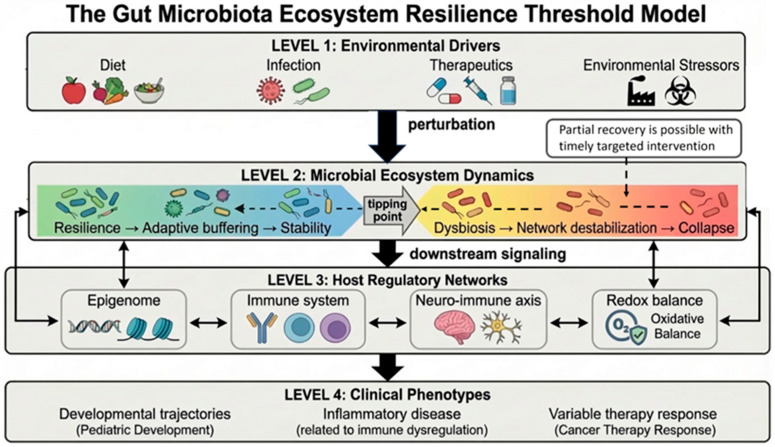
Conceptual representation of the Gut Microbiota Ecosystem Resilience Threshold Model. The schematic illustrates multi-level interactions between microbial functional capacity and host regulatory networks, including epithelial barrier integrity, immune calibration, neuro-immune communication, redox balance, and epigenetic modulation. Environmental perturbations (diet, infection, therapeutics, and environmental stressors) challenge system stability. When functional redundancy and host–microbe feedback loops are preserved, adaptive buffering maintains homeostasis. Exceeding a critical threshold results in cross-domain signaling amplification, inflammatory escalation, and systemic dysregulation; however, the post-threshold state may still retain partial reversibility under timely and targeted intervention. The model also highlights the host regulatory integration nodes as potential early-warning systems for identifying proximity to destabilizing transitions before overt clinical manifestation. Solid arrows denote direct mechanistic interactions; dashed arrows indicate feedback amplification, recovery pathways, and secondary regulatory loops.

Such multidimensional integration may enable early identification of destabilizing transitions before overt clinical manifestation, thereby shifting microbiome research from descriptive association toward predictive systems modeling (Figure 2). In this context, host regulatory integration nodes may also serve as early-warning and predictive systems, helping identify proximity to resilience failure and supporting intervention before irreversible cross-domain destabilization becomes clinically manifest.

## 9. Cross-Domain Amplification Principle

A defining feature of this framework is the concept of cross-domain amplification. Perturbations exceeding adaptive buffering at the microbial level propagate through host regulatory nodes, resulting in feedback loops that further destabilize microbial network stability. This bidirectional escalation distinguishes the present model from classical ecological resilience theory, which typically conceptualizes disturbance within self-contained ecosystems. In this sense, resilience failure is not a point event but an emergent property of network-level coupling collapse. Nevertheless, this collapse may still be modifiable, particularly if intervention occurs during early or intermediate stages of network destabilization, before self-reinforcing feedback loops become fully entrenched.

In this light, advancing microbiome research means not only identifying microbial signatures of disease, but restoring functional stability within host–microbe regulatory networks—a resilience-based perspective that supports integrative and predictive microbiome-informed strategies.
